# Dafachronic acid and temperature regulate canonical dauer pathways during *Nippostrongylus brasiliensis* infectious larvae activation

**DOI:** 10.1186/s13071-020-04035-z

**Published:** 2020-04-01

**Authors:** Katherine Omueti Ayoade, Faith R. Carranza, Woong Hee Cho, Zhu Wang, Steven A. Kliewer, David J. Mangelsdorf, Jonathan D. C. Stoltzfus

**Affiliations:** 1 Department of Pharmacology, University of Texas Southwestern Medical Center, Dallas, TX 75390 USA; 2 Department of Dermatology, University of Texas Southwestern Medical Center, Dallas, TX 75390 USA; 3 Department of Biology, Millersville University of Pennsylvania, Millersville, PA 17551 USA; 4 Department of Molecular Biology, University of Texas Southwestern Medical Center, Dallas, TX 75390 USA; 5 Howard Hughes Medical Institute, University of Texas Southwestern Medical Center, Dallas, Texas 75390 USA

**Keywords:** Nematode, Hookworm, Dauer, Infectious larva, Dafachronic acid, Insulin signaling, TGFβ, RNA-Seq

## Abstract

**Background:**

While immune responses to the murine hookworm *Nippostrongylus brasiliensis* have been investigated, signaling pathways regulating development of infectious larvae (iL3) are not well understood. We hypothesized that *N. brasiliensis* would use pathways similar to those controlling dauer development in the free-living nematode *Caenorhabditis elegans*, which is formally known as the “dauer hypothesis.”

**Methods:**

To investigate whether dafachronic acid activates the *N. brasiliensis* DAF-12 homolog, we utilized an *in vitro* reporter assay. We then utilized RNA-Seq and subsequent bioinformatic analyses to identify *N. brasiliensis* dauer pathway homologs and examine regulation of these genes during iL3 activation.

**Results:**

In this study, we demonstrated that dafachronic acid activates the *N. brasiliensis* DAF-12 homolog. We then identified *N. brasiliensis* homologs for members in each of the four canonical dauer pathways and examined their regulation during iL3 activation by either temperature or dafachronic acid. Similar to *C. elegans*, we found that transcripts encoding antagonistic insulin-like peptides were significantly downregulated during iL3 activation, and that a transcript encoding a phylogenetic homolog of DAF-9 increased during iL3 activation, suggesting that both increased insulin-like and DAF-12 nuclear hormone receptor signaling accompanies iL3 activation. In contrast to *C. elegans*, we observed a significant decrease in transcripts encoding the dauer transforming growth factor beta ligand DAF-7 during iL3 activation, suggesting a different role for this pathway in parasitic nematode development.

**Conclusions:**

Our data suggest that canonical dauer pathways indeed regulate iL3 activation in the hookworm *N. brasiliensis* and that DAF-12 may be a therapeutic target in hookworm infections.
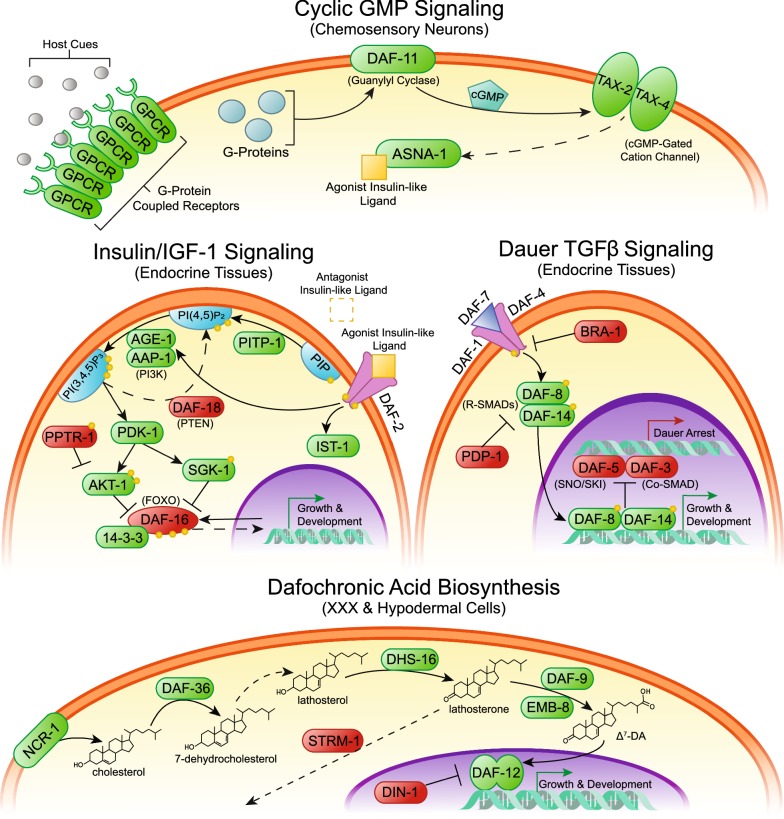

## Background

The human hookworms *Necator americanus* and *Ancylostoma duodenale* infect 500 to 700 million people globally, resulting in a significant disease burden in tropical and developing countries [1]. While the murine parasitic nematode *Nippostrongylus brasiliensis* is phylogenetically distinct from the hookworm species responsible for human disease [2], key aspects of the life-cycle are similar, including blood-feeding in the adult form [3] and a skin-penetrating infectious form that is developmentally arrested at the third larval stage (iL3) [4]. Since suitable non-human hosts do not exist for *N. americanus* and *A. duodenale* [5], *N. brasiliensis* is often utilized as a model for immunological studies of host responses to hookworm infection and can also be used to study mechanisms controlling hookworm development.

Hookworm larvae shed in host feces actively feed on bacteria during the first two larval stages before arresting their development as iL3. Although they are non-feeding, iL3 can persist in the soil for months due to stored lipids and are protected from desiccation by a plugged buccal cavity and retention of a sheath, which is the shed, but retained, L2 cuticle [4]. Once they encounter a host, iL3 rapidly penetrate the skin, exsheath, lose their buccal plug, and resume feeding, which is a hallmark of activation [6, 7]. In the canine hookworm *A. caninum*, iL3 require a combination of serum, glutathione and a host-like temperate of 37 °C for optimal resumption of feeding [8, 9]. While 37 °C is required for *N. americanus* exsheathment [10], only ~25% of *N. americanus* iL3 resume feeding at 37 °C in culture media and the percentage of larvae feeding does not increases with the addition of serum and/or glutathione [11]; thus, the requirements for optimal resumption of feeding are unknown in this species. In contrast, *N. brasiliensis* iL3 can activate, as evidenced by resumption of feeding, solely by incubation in culture media at 37 °C; this temperature-dependent activation is independent of serum and/or glutathione [12].

While the environmental cues that stimulate hookworm iL3 activation in *N. brasiliensis* have been investigated, the signaling pathways that transduce the stimuli are not well understood. A long-standing hypothesis in the field, known as the “dauer hypothesis”, posits that hookworm iL3 undergo arrest using similar genetic mechanisms as those controlling dauer arrest in the free-living nematode *Caenorhabditis elegans* [13, 14]. Four canonical dauer pathways regulate *C. elegans* dauer arrest: cyclic guanosine monophosphate (cGMP) signaling [15], dauer transforming growth factor β (TGFβ) signaling mediated by the DAF-7 ligand [16], insulin/insulin-like growth factor 1 (IGF-1) signaling (IIS) mediated by a complex mixture of agonistic and antagonistic insulin/IGF-1-like peptides (ILPs) [17] and a DAF-12 nuclear hormone receptor regulated by dafachronic acid steroid hormones that are synthesized by the cytochrome P450 DAF-9 [18, 19]. While some dauer pathways appear to have conserved roles in regulating iL3 development in parasitic nematodes, others do not [14].

In hookworms, pharmacological studies suggest that cGMP, IIS, and DAF-12 signaling pathways regulate iL3 development. In *A. caninum*, addition of a cGMP analog stimulates resumption of feeding in iL3 [20]. Furthermore, the muscarinic agonist oxotremorine can stimulate *A. caninum* iL3 recovery, while the muscarinic antagonist atropine can inhibit feeding; analogous studies in *C. elegans* suggest that this neuronal signaling is upstream of IIS [21]. In both *Ancylostoma* spp. and *N. brasiliensis*, the phosphatidylinositol 3-kinase inhibitor LY294002 can block resumption of feeding [12, 22], presumably by blocking IIS. In *A. caninum* and *N. brasiliensis*, iL3 activation can be blocked by the cytochrome P450 inhibitor ketoconazole [12, 23], presumably by inhibiting a DAF-9-like enzyme capable of producing dafachronic acids. Additionally, Δ7-dafachronic acid can stimulate iL3 feeding in *A. caninum*; however, LY294002-mediated inhibition of feeding cannot be rescued by addition of Δ7-dafachronic acid, suggesting that DAF-12 signaling may be parallel to IIS in the parasite [23]. While these studies suggest a conserved role for canonical dauer pathways in regulating both *C. elegans* dauer and hookworm iL3 development, studies of dauer TGFβ signaling are incongruous with this pattern. Transcripts of *A. caninum daf-7* are maximal in iL3, whereas *C. elegans daf-7* transcripts are downregulated during dauer arrest [24].

In this study, we systematically investigated the roles of canonical dauer pathways during iL3 activation in hookworms. To this end, we first demonstrated that dafachronic acids activate the *N. brasiliensis* DAF-12 nuclear hormone receptor. We then activated *N. brasiliensis* iL3 using both a host-like temperature of 37 °C and Δ7-dafachronic acid and subsequently quantified changes in transcript abundance for *N. brasiliensis* homologs in each of the canonical dauer pathways using RNA-Seq. We found that ILP-encoding transcripts are modulated by both temperature- and dafachronic acid-mediated activation, while *daf-7* transcripts decrease during iL3 activation. We also identified transcripts encoding cytochrome P450s that may be responsible for producing dafachronic acid in the parasite.

## Methods

### Maintenance of *Nippostrongylus brasiliensis*

*Nippostrongylus brasiliensis* was maintained in Sprague-Dawley rats, and iL3 were prepared/recovered from infected rat fecal cultures by the Baermann apparatus, as previously described [25]. Briefly, rat fecal culture was suspended in room temperature H_2_O or phosphate-buffered saline (PBS), allowing larvae to migrate into the media and gravity sediment. For experimental infections, 2000–4000 iL3 were prepared in 0.5 ml or less volume of media (PBS or water) and injected subcutaneously into a rat sedated with general anesthesia by inhalation of isoflurane.

### *In vitro* activation of *N. brasiliensis* iL3 and RNA extraction

Seven days after experimental infection, coprocultures were started on three consecutive days with sample A, B, and C representing fecal collection on days 7, 8, and 9, respectively. Rat feces were moistened with sterile water and mixed with coarsely granulated autoclaved charcoal; this mixture was placed on a moistened filter paper in a sterile Petri dish and coprocultures were incubated at 26 °C for two weeks. Subsequently, iL3 were prepared/recovered from rat coprocultures by the Baermann apparatus [25]. After the wash step, larvae were counted and axenized with Roswell Park Memorial Institute (RPMI) media supplemented with 25 mM HEPES and 1% penicillin/streptomycin for 40 min. Subsequently, iL3 were washed with sterile PBS and then resuspended in sterile RPMI media supplemented with L-Glutamine and 25 mM HEPES. Larvae from each sample were dispersed equally, in 10 ml aliquots, into each well of 6-well plates (Sample A = 7560 iL3/well, Sample B = 6066 iL3/well, and Sample C = 8400 iL3/well) and then half of the wells treated with vehicle (ethanol) and half with 10 μM (25S)-Δ7-dafachronic acid (CAS 949004-12-0) [26, 27]. Larvae were subsequently incubated at 20 °C, 26 °C or 37 °C for 24 hours (h).

Treatment was terminated after 24 h, larvae collected, culture media removed, and larvae resuspended in RNA-STAT60 (AMS Biotechnology Limited, Milton, UK); larvae were then snap-frozen using liquid nitrogen. Total RNA was extracted using a phenol-chloroform extraction method. Total RNA was DNase-I treated (Roche, Basel, Switzerland) and subsequently purified using an RNeasy purification kit (Qiagen, Hilden, Germany). Total RNA was analyzed on a Bioanalyzer 2100 (Agilent, Santa Clara, USA) to determine level of degradation; only RNA with a RIN Score of 9.0 or higher was used. Total RNA concentration was determined using a Qubit fluorometer (Thermo Fisher Scientific, Waltham, USA).

Resumption of feeding of iL3 was assessed by collecting iL3 from coprocultures incubated at 26 °C for seven days by Baermann funnel. The iL3 were washed and resuspended in RPMI as previously described. Approximately 100 iL3 were transferred to each well of a 96-well plate and incubated with either vehicle (ethanol) or Δ7-dafachronic acid at 22 °C, 26 °C or 37 °C for 22 h. Subsequently, fluorescein isothiocyanate-bovine serum albumin (FITC-BSA) at 50 mg/ml was added to each well and cultures incubated for an additional 2 h. The iL3 were then transferred to tubes, washed three times with PBS, and presence/absence of ingested FITC-BSA observed by fluorescent microscopy.

### RNA-Seq

Libraries were prepared using 1 μg of total DNase-treated RNA using the TruSeq Stranded Total RNA LT Sample Prep Kit (Illumina, San Diego, USA), with the total RNA depleted of rRNA before strand-specific cDNA synthesis. Adapter-ligated libraries were polymerase chain reaction (PCR) amplified and purified with AmpureXP beads, then validated again on the Tapestation 4200. Individual libraries were normalized and pooled using the Qubit and sequenced on the NextSeq 500 (Illumina) using V2.5 reagents. Raw data were then de-multiplexed and converted to fastq files using bcl2fastq v2.17 (Illumina); raw reads are available under BioProject ID PRJNA574186.

### Read processing, mapping, and *de novo* assembly

Fastqc files were inspected using FastQC v0.11.7 [28] and trimmed of index sequences and low-quality bases using Trimmomatic v.0.38 [29] and the options ILLUMINACLIP:2:30:10:3, LEADING:5, TRAILING:5, SLIDINGWINDOW:4:5, and MINLEN:50; removal of index sequences was confirmed using FastQC. Trimmed reads were then depleted of contaminating rRNA sequences using bbduk.sh (BBMap v38.06) [30] and the following reference sequences: a *N. brasiliensis* rDNA scaffold constructed using *18S* rDNA (GenBank: AJ920356), ITS1 (AY332646), ITS2 (AY333380), and *28S* rDNA (AM039748) together with *de novo* assembled transcripts to deduce ETS and *5.8S* rDNA sequences, the *5S* rDNA sequence with ETS sequences identified with a BLAST search using the *Haemonchus contortus 5S* rDNA (HCU32122) and mtDNA sequences (AP017690). Processed reads were then mapped to the *N. brasiliensis* genome (WormBaseParaSite v9; BioProject PRJEB511) using HISAT2 v2.1.0 and the options –dta, –rna-strandness RF, –max-intronlen 50000 [31] and BAM files generated using SAMtools v1.7 [32]. *De novo* assembly was performed by first merging trimmed forward and reverse reads from all of the samples in this study using bbmerge.sh (BBMap v38.06) with maxstrictness parameters and trimmed to q-score > 20; transcripts were then *de novo* assembled using Trinity v2.3 [33, 34] on Galaxy [35]. *De novo* assembled transcripts are available under BioProject ID PRJNA574186.

### Transcript identification

*Caenorhabditis elegans* and *H. contortus* homologs were used as local BLAST search queries against the *N. brasiliensis* genome using Geneious v10.2.2 (Biomatters Ltd., Auckland, New Zealand) to identify contigs with homology. Hits were manually annotated in Geneious using aligned RNA-Seq reads in the Integrated Genome Viewer v2.4.10 [36, 37] as a guide. When transcripts were split between > 1 genomic contig/scaffold, *de novo* assembled RNA-Seq reads were used as a guide to reconstruct full-length sequences when possible. Annotated coding sequences (Additional file 1: Data S1, Additional file 2: Data S2) were used to predict *N. brasiliensis* protein sequences (Additional file 3: Data S3), which were used as BLAST search queries to confirm gene identity. Protein alignments using ClustalW and neighbor-joining phylogenetic trees in Geneious were used to resolve the identity of similar *N. brasiliensis* proteins, including cytochrome P450s and SMAD proteins in the TGFβ pathway. Insulin-like peptides were identified using BLAST searches of both the *N. brasiliensis* genome and *de novo* assembled transcriptome translated in all six reading frames; BLAST hits were manually screened for conserved cystine residues with appropriate spacing, a conserved glycine after the first cystine, conserved hydrophobic residues, and a predicted signal peptide.

Alignment of insulin-like peptide protein sequences was performed using ClustalW in Geneious and manually adjusted; signal peptides were predicted using SignalP-5.0, and key residues were identified by manual inspection [38, 39]. Alignment of the ligand-binding domains of the predicted DAF-7 encoding transcripts from *C. elegans* (GenBank: NP497265), *H. contortus* (GenBank: ACQ84508), *N. americanus* (GenBank: XP013301606), and *N. brasiliensis* was performed using ClustalW in Geneious.

### Cloning and expression of *N. brasiliensis* cytochrome P450 homologs

A pCMX-derived expression vector [40] that incorporates a C-terminal hemagglutinin (HA) tag was created by digesting the plasmid with *Bam*HI and *Nhe*I (New England BioLabs, Ipswich, USA), and the short insert, created by annealing the oligonucleotides BamHI-HA-NheI-F and BamHI-HA-NheI-R (sequences in Additional file 4: Data S4), was ligated into the digested vector using T4 DNA ligase (NEB); the reaction mixture used to transform chemically competent *Escherichia coli* Turbo cells (NEB) using the manufacturer’s instructions. The insert of the recombinant plasmid was sequenced using the primers pCMV-1F and pCMX-2R (sequences in Additional file 4: Data S4); this resulted in the plasmid pJS96, which was used for expression of C-term HA-tagged *N. brasiliensis* cytochrome P450 transcripts.

Total RNA was purified from *N. brasiliensis* iL3 that were either treated with vehicle or dafachronic acid and treated with DNase; subsequently, cDNA was synthesized from 290 ng (vehicle-treated) or 540 ng (dafachronic acid-treated) DNase-treated total RNA using ProtoScript II and an oligo dT primer according to the manufacturer’s instructions (NEB). Coding sequences (excluding the stop codons) of *Nbr-cyp-14a7*, *Nbr-cyp-22a1*, *Nbr-cyp-32a1*, *Nbr-cyp-42a1*, and *Nbr-cyp-43a1* were amplified using Q5 DNA polymerase (NEB) and primers with restriction sites (sequences in Additional file 4: Data S4). PCR products were purified by polyethylene glycol (PEG) precipitation and were double-digested with either *Kpn*I or *Xho*I and *Bam*HI (NEB); the pJS96 vector was similarly digested and also treated with rSAP (NEB). Digested PCR products were purified using the Monarch PCR and DNA Cleanup Kit (NEB), while the digested pJS96 vector was purified *via* gel extraction using the Monarch Gel Extraction Kit (NEB); subsequently, inserts were ligated using T4 DNA ligase (NEB) and the manufacturer’s instructions. Ligation reaction products were used to transform chemically competent *E. coli* Turbo cells (NEB). Plasmids were isolated from single colonies and sequenced using the primers pCMV-1F and pCMX-2R, resulting in the plasmids pJS160 (*Nbr-cyp-14a7*), pJS161 (*Nbr-cyp-22a1*), pJS162 (*Nbr-cyp-32a1*), pJS163 (*Nbr-cyp-42a1*), and pJS164 (*Nbr-cyp-43a1*), which include a CMV promoter driving expression of a C-terminally HA-tagged *N. brasiliensis* coding sequence with an SV40 terminator.

### Cloning *N. brasiliensis daf-12*

Expression vectors containing *A. caninum* DAF-12 and *C. elegans* DAF-12, as well as the pCMX parent vector, have been previously described [18, 23]. To construct a similar expression vector for *N. brasiliensis* DAF-12, the 5’-end was amplified from iL3-derived cDNA using the SMARTer RACE 5’/3’ Kit (Takara Bio USA Inc., Mountain View, USA) and the 3’-end was amplified from iL3-derived cDNA using the 3’ RACE System for Rapid Amplification for cDNA Ends (Invitrogen, Carlsbad, USA); the 5’- and 3’-ends were fused by overlap-extension PCR (primer sequences in Additional file 4: Data S4). The PCR product was inserted into the pCMX expression vector and the insert verified by sequencing.

### *In vitro* DAF-12 reporter assays

Co-transfection and luciferase reporter assays were performed in human embryonic kidney (HEK) 293 cells, as previously described [23]. Briefly, co-transfections in HEK293 cells were performed in 96-well plates using 50 ng of luciferase reporter, 20 ng of CMX-β-galactosidase reporter, and either 15 ng of CMX-DAF-12 receptor expression plasmid or CMX vector control plasmid. Reporter plasmids were constructed by inserting DAF-12 response elements and their 10-bp genomic flanking sequences into a TK-luc reporter plasmid. Eight hours post-transfection, cells were treated with either vehicle (ethanol), (25S)-Δ4-dafachronic acid (CAS 23017-97-2), or Δ7-dafachronic acid; luciferase and β-galactosidase activities were then measured 16 h later. Relative luciferase units (RLU) were normalized to β-galactosidase activity. This was repeated in triplicate.

### Transcript abundance quantification and differential expression analysis

*Nippostrongylus brasiliensis* transcripts were predicted for each sample using StringTie v.1.3.4d [31] and the options: –rf, -f 0.15, and a GTF file containing the manually annotated dauer pathway transcripts. Resulting GTF files were subsequently merged using StringTie –merge, and the resulting merged GTF annotation file manually corrected (Additional file 5: Data S5). Read counts for *N. brasiliensis* transcripts were quantified for each sample using StringTie; only reads that mapped to transcripts in the merged annotation file were used for quantification. Read counts for genes were calculated using the *prepDE.py* script with the StringTie package. Differential expression analysis and transcript quantification was performed using EdgeR v3.24.1 [41], using trimmed mean of M values (TMM) normalization (Additional file 6: Data S6). For significance, a minimum log_2_ fold change of one, a *P*-value adjusted threshold of < 0.05 and *P*-value adjustment method of Benjamini & Hochberg for false-discovery-rate were used (Additional file 7: Data S7).

### Data visualization

Abundances of insulin-like peptide-encoding transcripts and the DAF-7-encoding transcript were plotted using EdgeR-calculated counts per million reads mapped (CPM) values in GraphPad Prism v.7.04 (GraphPad Software, Inc., San Diego, USA). Pharmacological studies of dafachronic acid-mediated activation of DAF-12 were plotted in GraphPad Prism. The heat map depicting abundance of transcripts in the dauer TGFβ pathway was constructed using row-scaled CPM values in *heatmap.2* (gplots v3.0.1.1) in R; similarly, the heat map depicting abundance of cytochrome P450-encoding transcripts was constructed using row-scaled CPM values in *heatmap.2* and a dendrogram constructed using the Manhattan method for calculating clustering distance. When transcripts for a single gene were split over multiple contigs/scaffolds, the fragment with the most abundant CPM was plotted.

## Results

### *Nippostrongylus brasiliensis* DAF-12 is activated by dafachronic acid

**Fig. 1:**
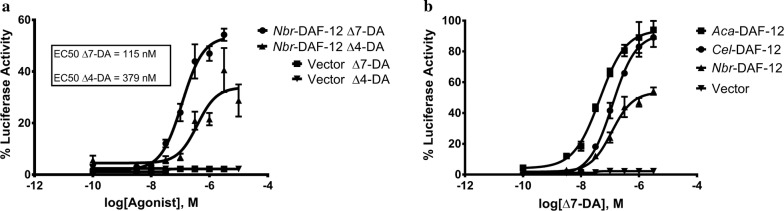
Dafachronic acid is an agonist of *N. brasiliensis* DAF-12. **a***In vitro*, both Δ7-dafachronic acid (Δ7-DA) and Δ4-dafachronic acid (Δ4-DA) act as agonists for *N. brasiliensis* nuclear hormone receptor *Nbr*-DAF-12, although Δ4-dafachronic acid is a weaker agonist. **b** In comparison to other clade V/clade 9 nematode species, *N. brasiliensis* DAF-12 responds more weakly to Δ7-dafachronic acid than DAF-12 from either the canine hookworm *A. caninum* (*Aca*-DAF-12) or the free-living *C. elegans* (*Cel*-DAF-12). **a**, **b** Co-transfection assays were performed in HEK293 cells in biological triplicate; luciferase activity was quantified in relative light units and the maximal reading observed scaled to 100%. Data represent the mean ± standard deviation of triplicate assays

In *C. elegans*, the nuclear hormone receptor DAF-12 is epistatic to cGMP signaling, IIS, and dauer TGFβ signaling [42, 43] and is regulated by a class of steroid ligands known as dafachronic acids [18]. Dafachronic acid induces dauer recovery in *C. elegans* [18] and iL3 activation in both *H. contortus* and *Strongyloides stercoralis* [23, 44–46]. Furthermore, Δ7-dafachronic acid activates the ligand-binding domain of DAF-12 in *Ancylostoma* spp. and *N. americanus* [23, 47]. To determine whether the *N. brasiliensis* DAF-12 homolog is similarly activated by dafachronic acid, we expressed *Nbr-daf-12* in HEK293 cells along with a *daf-12* response element driving a luciferase reporter. Similar to other species, we found that *Nbr*-DAF-12 is activated by Δ7-dafachronic acid (EC50 = 115 nM) and to a lesser extent by Δ4-dafachronic acid (EC50 = 379 nM) (Fig. 1a). We then compared the Δ7-dafachronic acid response of *Nbr*-DAF-12 to the response of DAF-12 homologs from *A. caninum* and *C. elegans*. We found that *Nbr*-DAF-12 responds more weakly to Δ7-dafachronic acid than DAF-12 from other species (Fig. 1b).

### RNA-Seq of iL3 activated by temperature or dafachronic acid

**Fig. 2:**
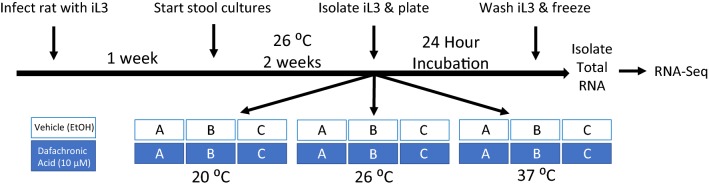
Culture conditions of *N. brasiliensis* iL3 and RNA-Seq experimental design. Rats were experimentally infected with iL3; after patency (1 week), stool was collected for three consecutive days and larvae cultured for two weeks at 26 °C. Subsequently, iL3 were isolated and cultured for 24 h with either Δ7-dafachronic acid (DA) or vehicle (ethanol) at 20 °C, 26 °C or 37 °C for both treatments. Each condition was performed in triplicate, with sample A derived from the stool culture on the first day of collection, sample B on the second day, and sample C on the third day. Total RNA was then isolated from iL3 and used to construct RNA-Seq libraries

While pharmacological studies have demonstrated roles for cGMP signaling [20], IIS [22, 48], and dafachronic acid signaling [23] in hookworm activation, transcriptional regulation of dauer signaling pathways during *N. brasiliensis* activation has not been studied. To determine how these pathway components are regulated during iL3 activation and the role of dafachronic acid signaling in this process, we utilized RNA-Seq to examine differences in transcript abundance in *N. brasiliensis* iL3 activated by temperature and/or dafachronic acid (Fig. 2). Since *N. brasiliensis* iL3 can be activated solely by incubation at 37 °C in RPMI media [12], we sought to compare differences in transcript abundance in iL3 cultured at 20 °C or 26 °C to iL3 cultured at 37 °C, as iL3 only resume feeding when cultured in the vehicle control at 37 °C (Additional file 8: Table S1). Since Δ7-dafachronic acid stimulates iL3 resumption of feeding at non-permissive temperatures (Additional file 8: Table S1), we also compared transcript abundances in iL3 activated by dafachronic acid at the non-permissive temperatures of 20 °C and 26 °C, as well as the permissive temperature of 37 °C, to iL3 in temperature-matched vehicle controls.

**Fig. 3:**
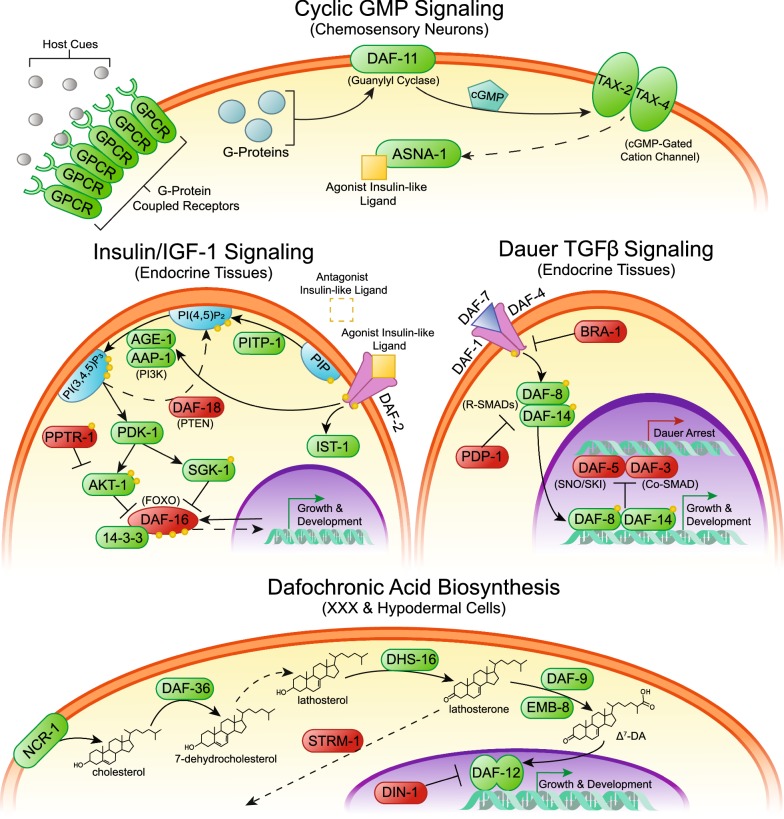
Hypothesized dauer signaling during *N. brasiliensis* iL3 activation. Using reciprocal BLAST searches, we identified the homologs diagrammed above of canonical dauer pathway signaling molecules in the *N. brasiliensis* genome and *de novo* assembled transcriptome. We hypothesize that GPCRs in the chemosensory neurons receive cues from the host such as temperature and host-specific chemicals resulting in production of ligands for insulin/IGF-1 and dauer TGFβ signaling pathways. Additionally, we hypothesize that a dafachronic acid biosynthesis pathway upregulates production of this steroid hormone. Together, we hypothesize that activation of the cognate transcription factors in these pathways commit the infectious larvae to reproductive growth and development. Green boxes denote proteins that stimulate growth and development, while red boxes denote proteins that stimulate dauer arrest (Adapted from [39])

In order to identify canonical dauer signaling pathway components in *N. brasiliensis*, we performed reciprocal BLAST searches of the *N. brasiliensis* genome using *C. elegans* or *H. contortus* protein sequences as queries, because both species are clade V/clade 9 nematodes and dauer pathway homologs have been identified in *H. contortus* [49, 50]. Since the *N. brasiliensis* genome assembly is highly fragmented, resulting in some genes being split amongst two or more contigs/scaffolds, we also utilized *de novo* assembled transcripts to manually reconstruct coding sequences of each gene. Together, we were able to identify the key signaling pathway components in each of the four canonical dauer pathways in *N. brasiliensis* (Fig. 3). We then sought to examine differences in the regulation of key transcripts in these signaling pathways during the initial phase of iL3 activation.

### cGMP signaling is modulated during iL3 activation

We hypothesized that during *N. brasiliensis* iL3 activation, host-like environmental cues would result in increased cGMP signaling, but that transcript abundance of pathway components would not change or would moderately increase in response to increased flux through this pathway. Interestingly, we observed a significant decrease in the abundance of *Nbr-daf-11* and *Nbr-tax-4* transcripts during iL3 activation with temperature (37 °C) or dafachronic acid (20 °C) in comparison to iL3 at 20 °C in the control treatment (Additional file 9: Figure S1). We also observed a trend towards a decrease in *Nbr-tax-2* transcripts, but the fold-change was less than our cut-off of two for statistical significance.

### IIS increases during iL3 activation

**Fig. 4:**
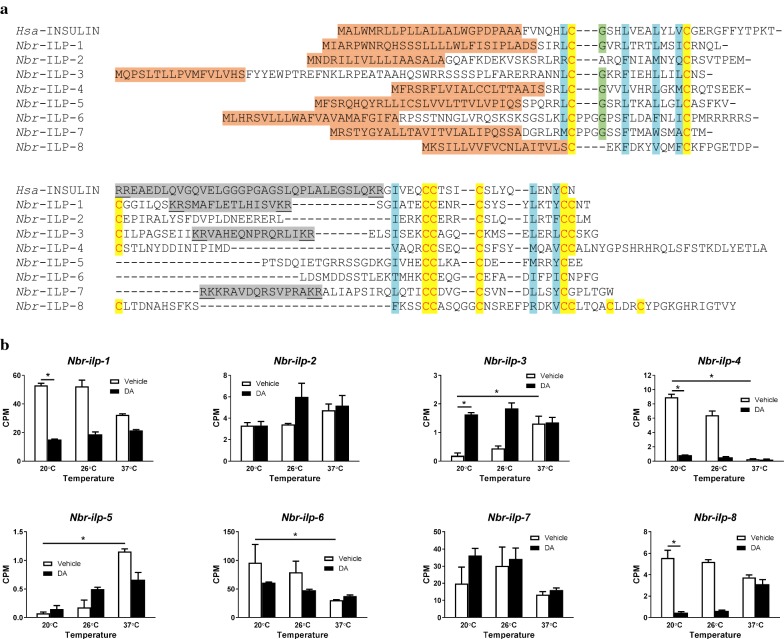
Insulin-like peptides are regulated by both temperature and dafachronic acid during *N. brasiliensis* iL3 activation. **a** Protein alignment of eight predicted *N. brasiliensis* insulin-like peptides (ILPs) with human insulin. Predicted signal peptides are highlighted in orange; conserved cysteine residues predicted to form disulfide bonds are in red letters and highlighted in yellow; a conserved glycine residue is highlighted in green; hydrophobic residues needed for helix formation are highlighted in blue; and predicted C-peptides are highlighted in gray with predicted cleavage sites underlined. *Nbr-*INS-1 through -4 have β type architecture; *Nbr-*INS-5 through -7 have γ type architecture; and *Nbr-*INS-8 has δ type architecture. **b** Transcript abundances were determined for the coding region of eight genes encoding insulin-like peptides, *Nbr-ilp-1* through *-8*, in iL3 treated with either Δ7-dafachronic acid (DA) or the vehicle control (ethanol) for 24 h at 20 °C, 26 °C or 37 °C. TMM-normalized transcript abundance was plotted as the mean counts per million (CPM) for each condition; error bars represent the SEM. Statistical significance was evaluated between 20 °C vehicle and 20 °C DA conditions and also between 20 °C vehicle and 37 °C vehicle conditions; *, fold-change > 2.0, FDR-adjusted *P*-value < 0.05

Since changes in IIS typically result from changes in ligand concentration rather than number of molecules in the signaling pathway itself, we hypothesized that iL3 activation in *N. brasiliensis* would be accompanied by an increase in transcripts encoding ILP agonists and a decrease in antagonists, while transcripts encoding signaling pathway components would be largely unchanged. To this end, we first identified the ILPs encoded by the *N. brasiliensis* genome through BLAST searches of both the genome and *de novo* assembled transcriptome. We found a total of eight ILPs (Fig. 4a), although others may be present but unidentified since transcriptomic data from other *N. brasiliensis* developmental stages are not available. Four of these peptides, *Nbr*-ILP-1 through -4, have β-type architecture, while three, *Nbr-*ILP-5 through -7, have γ-type architecture; we did not identify any peptides with α-type architecture. We also identified a single peptide, *Nbr-*ILP-8, with δ-type architecture, which has only been previously reported in the parasitic nematode *S. stercoralis* [39].

Based on phylogenetic analysis, the predicted peptide *Nbr*-ILP-1 is most similar to the *C. elegans* β-type INS-1, which acts as an antagonist [38, 51–53]; we therefore hypothesized that *Nbr*-ILP-1 also acts as an antagonist. To test this hypothesis, we examined the regulation of *Nbr-ilp-1* transcript abundance during iL3 activation (Fig. 4b). We found a significant reduction in *Nbr-ilp-1* transcripts in iL3 stimulated with dafachronic acid at 20 °C in comparison to the vehicle-treated control. Additionally, both *Nbr*-ILP-6 and *Nbr*-ILP-7 predicted peptides contain a PPG motif, which is present in the γ-type *C. elegans* antagonists *Cel*-INS-17 and *Cel*-INS-18 [38, 53, 54]. Based on their structure, we hypothesized that *Nbr*-ILP-6 and *Nbr*-ILP-7 would also be antagonists, with *Nbr*-ILP-6 more similar to *Cel*-INS-17 due to the absence of a C-peptide and *Nbr*-ILP-7 more similar to *Cel*-INS-18 due the presence of a C-peptide that is predicted to be cleaved. Therefore, we examined the regulation of *Nbr-ilp-6* and *-7* transcript abundance during iL3 activation (Fig. 4b). While temperature-dependent activation resulted in a significant decrease in *Nbr-ilp-6* transcripts at 37 °C in comparison to the 20 °C control, changes in *Nbr-ilp-7* transcript abundance were more equivocal. Although changes in transcript abundance during iL3 activation suggest that *Nbr-ilp-4* and *-8* (whose expression was downregulated) may also act as antagonists, and that *Nbr-ilp-2*, *-3*, and *-5* (whose expression was upregulated) may act as agonists, we do not have an independent means of testing these hypotheses.

The majority of transcripts encoding the signaling molecules in the IIS pathway are not differentially regulated by activation *via* either temperature or dafachronic acid (Additional file 10: Figure S2). However, transcripts for *Nbr-akt-1*, *Nbr-pitp-1*, and *Nbr-daf-16* each decreased significantly in both iL3 stimulated with dafachronic acid at 20 °C and temperature at 37 °C, in comparison to the 20 °C control. Conversely, *Nbr-sgk-1* transcripts increased significantly in both iL3 stimulated with dafachronic at 20 °C and temperature at 37 °C, in comparison to the 20 °C control. Taken together, these data support the notion that the IIS pathway is present and switched on during iL3 activation.

### Dauer TGFβ signaling decreases during iL3 activation

**Fig. 5:**
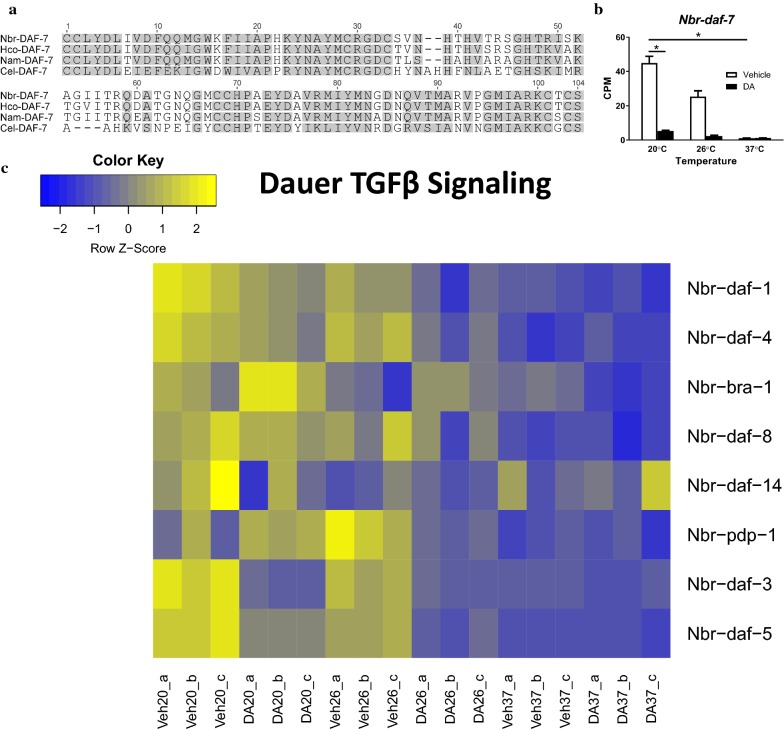
Dauer TGFβ signaling is downregulated during *N. brasiliensis* iL3 activation. **a** Predicted protein alignment of the ligand domains of DAF-7-like TGFβ ligands from *N. brasiliensis* (Nbr-DAF-7), *H. contortus* (Hco-DAF-7), *N. americanus* (Nam-DAF-7), and *C. elegans* (Cel-DAF-7). Gray indicates similar amino acids. **b** Transcript abundance was determined for the coding region of *Nbr-daf-7* in iL3 treated with either Δ7-dafachronic acid (DA) or the vehicle control (ethanol) for 24 h at 20 °C, 26 °C or 37 °C. TMM-normalized transcript abundance was plotted as the mean counts per million (CPM) for each condition; error bars represent the SEM. Statistical significance was evaluated between 20 °C vehicle and 20 °C DA conditions and also between 20 °C vehicle and 37 °C vehicle conditions; *, fold-change > 2.0, FDR-adjusted *P*-value < 0.05. **c** Heat map depicting differences in transcript abundance in iL3 treated with either Δ7-dafachronic acid (DA) or the ethanol vehicle control (Veh) for 24 h at 20 °C, 26 °C or 37 °C. Transcript abundance was z-scaled for each gene; biological replicates are indicated with a, b or c

In *C. elegans*, *daf-7*, *dbl-1*, *unc-129*, and *tig-2* all encode TGFβ ligands; however, only DAF-7, which signals through the DAF-1 and DAF-4 heterodimeric receptor, has a clear role in controlling dauer development [16]. To identify the *N. brasiliensis* homolog of *daf-7*, we utilized both protein alignment and phylogenetic analyses, which are summarized in Figure 5a. Since the dauer TGFβ signaling pathway ligand, DAF-7, promotes reproductive growth and development in *C. elegans* [55, 56], and *Cel-daf-7* transcripts decrease during dauer arrest [56, 57], we hypothesized that transcripts of the *N. brasiliensis* homolog of *daf-7* would increase in abundance during iL3 activation. Surprisingly, we found the opposite: *Nbr-daf-7* transcripts decreased more than five-fold during iL3 activation at 37 °C in comparison to the 20 °C control and more than three-fold during activation with dafachronic acid at 20 °C in comparison to the 20 °C control (Fig. 5b).

Similar to IIS, we hypothesized that transcripts encoding dauer TGFβ signaling components would be largely unchanged during iL3 activation. Most pathway components had only small changes in transcript abundance; however, the Co-Smad and Sno/Ski-like negative regulators encoded by *Nbr-daf-3* and *Nbr-daf-5*, respectively, were significantly downregulated during iL3 activation (Fig. 5c). In iL3 activated at 37 °C, both *Nbr-daf-3* and *Nbr-daf-5* transcript abundances significantly decreased in comparison to the 20 °C control. For *Nbr-daf-3*, transcript abundance also decreased significantly with dafachronic acid activation at 20 °C in comparison to the 20 °C control, and although *Nbr-daf-5* also decreased, the fold-change was less than our cut-off of two for statistical significance. Together, these data suggest the dauer TGFβ signaling pathway is present and that DAF-7 signaling is downregulated during iL3 activation.

### A *N. brasiliensis* DAF9-like cytochrome P450 transcript increases during iL3 activation

In *C. elegans*, *daf-9* encodes a cytochrome P450 enzyme that produces dafachronic acid [18], with *daf-9* transcripts upregulated during growth and development as well as during dauer exit [58, 59]. Thus, we sought to identify which gene(s) may be responsible for production of dafachronic acid in *N. brasiliensis*. First, we utilized the *N. brasiliensis* draft genome and our *de novo* assembled RNA-Seq reads to identify transcripts predicted to encode cytochrome P450 enzymes. In total, we identified 44 transcripts that encode a predicted cytochrome P450 domain, which we classified by standard nomenclature by similarity to other characterized cytochrome P450s [45, 60]. *Nbr*-CYP22A1 was most similar phylogenetically to *Cel*-DAF-9 (Additional file 11: Figure S3).

**Fig. 6:**
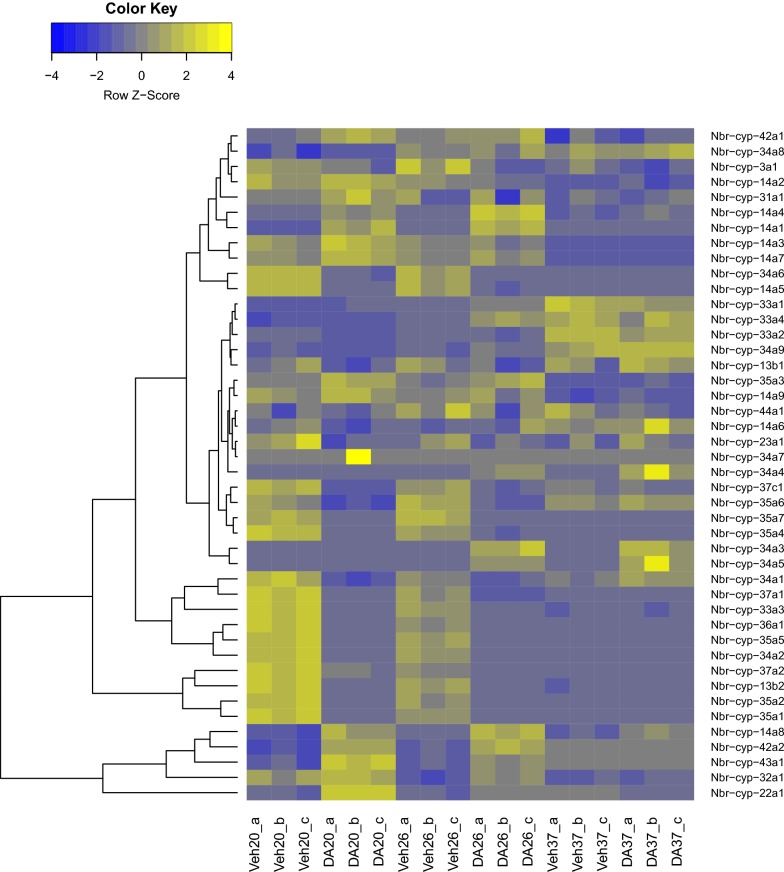
Dafachronic acid and temperature upregulate the abundance of a subset of *N. brasiliensis* cytochrome P450-encoding transcripts. Heat map depicting differences in transcript abundance of genes encoding cytochrome P450s in iL3 treated with either Δ7-dafachronic acid (DA) or the ethanol vehicle control (Veh) for 24 h at 20 °C, 26 °C or 37 °C. Transcript abundance was z-scaled for each gene and a dendrogram constructed to group genes by similarity of transcript regulation. Biological replicates are indicated with a, b or c

Since increases in dafachronic acid biosynthesis in *C. elegans* result in upregulation of *daf-9* transcripts *via* a DAF-12-mediated positive feedback loop [61], we reasoned that *N. brasiliensis* iL3 activation either by temperature or dafachronic acid would result in an increase in transcripts encoding the cytochrome P450(s) responsible for dafachronic acid production in the parasite. Using our RNA-Seq data, we identified a subset of *N. brasiliensis* cytochrome P450 transcripts that increase in abundance during iL3 activation, which includes *Nbr-cyp22a1* (Fig. 6, Additional file 12: Data S8). To determine whether members of this group are capable of dafachronic acid production *in vitro*, we cloned *Nbr-cyp-14a7*, *Nbr-cyp-22a1*, *Nbr-cyp-42a1*, and *Nbr-cyp-43a1* and attempted to express these *in vitro*, similar to previous studies in *C. elegans* [18]. However, we were unable to reliably express these *N. brasiliensis* cytochrome P450 proteins in HEK293 cells, similar to difficulties we have encountered with expressing *S. stercoralis* cytochrome P450 sequences (data not shown).

We also examined differences in transcript abundance of other components of the proposed dafachronic acid biosynthetic pathway during *N. brasiliensis* iL3 activation (Additional file 13: Figure S4). We observed a significant increase in *Nbr-daf-36* transcripts, predicted to encode a Rieske-like oxygenase, in iL3 activated at 37 °C in comparison to the 20 °C control, and we observed a significant increase in *Nbr-dhs-16* transcripts, predicted to encode a short-chain dehydrogenase, in iL3 activated at 37 °C or with dafachronic acid at 20 °C, both in comparison to iL3 incubated at 20 °C. We also observed a significant decrease in *Nbr-daf-12* transcripts at 37 °C in comparison to the 20 °C control. Altogether, these data support our hypothesis that dafachronic acid promotes iL3 activation in *N. brasiliensis*.

## Discussion

Nematode parasitism of plants and animals is common and is thought to have arisen at least 15 times over the course of the phylum’s evolutionary history [50], suggesting that nematodes have specific developmental features that aid the transition to parasitism [62]. A longstanding hypothesis in the field, known as the “dauer hypothesis”, posits that the dauer stage of free-living nematodes aided the transition to parasitism [14]. While the genes that control dauer development are well understood in the free-living nematode *C. elegans* [15], the genes that control development of infectious larvae in parasitic nematodes are less clear, since IIS and DAF-12 signaling pathways appear to have similar functions, but TGFβ signaling appears to function differently [14]. In this study, we sought to characterize homologs of the four canonical dauer signaling pathways in *N. brasiliensis* and determine whether these pathways play a role in iL3 activation.

To further our understanding of how dafachronic acid regulates hookworm iL3 activation, we first investigated the DAF-12 nuclear hormone receptor. In this study, we cloned the *N. brasiliensis* gene encoding DAF-12 and demonstrated that it responds to both Δ4- and Δ7-dafachronic acid. Similar to other parasitic nematodes [23], *Nbr*-DAF-12 responds more strongly to Δ7-dafachronic acid than to Δ4-dafachronic acid, suggesting that Δ7-dafachronic acid may be the native ligand, or more similar to the native ligand(s), in the parasite. To determine how dafachronic acid regulates iL3 activation and compare this to temperature-mediated activation, we utilized RNA-Seq to examine changes in transcript abundance in the four canonical dauer signaling pathways using these two stimuli.

We were able to identify all major components in these four pathways and reconstruct transcripts for nearly all components in spite of the highly fragmented state of the *N. brasiliensis* genome. Most dauer signaling pathway genes found in *C. elegans* (clade 9A) were also present in *N. brasiliensis* (clade 9B) [2], with the exception of the number of ILPs, with 40 present in *C. elegans* [17] and eight identified in *N. brasiliensis* in this study. A paucity of ILPs in the ruminant parasitic nematode *H. contortus* (clade 9B) [49], as well as in the more distantly related human parasitic nematode *S. stercoralis* (clade 10B) [39], suggests that the number of ILPs have expanded in the *Caenorhabditis* lineage and are found in more modest numbers in parasitic nematodes. Conservation of the components in the other dauer signaling pathways is congruous with both *N. brasiliensis* and *C. elegans* having descended from a common ancestor in the clade V/clade 9 lineage [2, 50].

Although components of the cGMP signaling pathway are conserved in *N. brasiliensis*, the role they play during iL3 activation is less clear. While application of the exogenous cGMP analog 8-bromo-cGMP at 37 °C stimulates iL3 to resume feeding in *A. caninum* [20] as well as in the distantly related *S. stercoralis* [44], addition of 8-bromo-cGMP does not stimulate *N. brasiliensis* iL3 to begin feeding at 20 °C [12]. Since temperature alone is sufficient to induce feeding in *N. brasiliensis* iL3 [12], and feeding studies of either *A. caninum* or *S. stercoralis* using 8-bromo-cGMP at low temperatures have not been performed, it is difficult to determine whether there are differences in cGMP pathway activation between *N. brasiliensis* and other soil-transmitted helminths. Furthermore, we are unaware of a study directly examining changes in cGMP levels during iL3 activation in a parasitic nematode and whether this correlates with changes in transcript abundance in signaling pathway components. Thus, we are unable to fully ascertain the biological relevance of the decreases in transcript abundance of *daf-11*, *tax-2*, and *tax-4* during iL3 activation in *N. brasiliensis*, although similar decreases have been observed in iL3 activation in *H. contortus* [49] and to a lesser extent in *S. stercoralis* [39] and *T. canis* [63]. Although specific homologs were not identified, receptor-gated ion channels are downregulated in *A. ceylanicum* during early infection of the golden hamster host [64].

Both pharmacological studies and our transcriptomic data support a role for increased IIS in hookworm iL3 activation. Inhibition of phosphatidylinositol 3-kinases blocks iL3 activation in hookworms [12, 22, 23] and *S. stercoralis* [65]. Based on our structural comparisons, we identified three ILP antagonists: *Nbr*-ILP-1, *Nbr*-ILP-6, and *Nbr*-ILP-7. Both *Nbr-ilp-1* and *Nbr-ilp-6* were downregulated during iL3 activation, similar to downregulation of putative ILP antagonists in *H. contortus* [46, 49] and *S. stercoralis* [39, 44] during iL3 activation. Our transcriptomic data also suggest that *Nbr-ilp-2*, *Nbr-ilp-3*, and *Nbr-ilp-5* may act as agonists during iL3 activation. In future studies, determining whether upregulation of these putative IIS agonists is a key driver of iL3 activation in hookworms may be particularly informative in understanding mechanisms of pathogenesis. Together, these data support our hypothesis that increased IIS accompanies iL3 activation in both hookworms and other parasitic nematodes [66, 67], playing a similar role in larval growth and development as in *C. elegans* [17].

In *C. elegans*, *daf-7* mutants have a dauer constitutive phenotype [55] and *Cel-daf-7* expression is severely diminished in the dauer larva [56], suggesting that dauer TGFβ signaling is at a minimum in dauer larvae and increases upon activation. In direct contrast, transcripts for *Nbr-daf-7*, which are predicted to encode the dauer TGFβ ligand, are abundant in iL3 and suppressed by either dafachronic acid- or temperature-mediated activation. Downregulation of the DAF-7 TGFβ ligand suggests that dauer TGFβ signaling is suppressed during *N. brasiliensis* iL3 activation. Since similar observations have been made in a variety of clade IV/clade 10 and clade V/clade 9 parasitic nematodes [24, 39, 49, 57, 68], the upregulation of dauer TGFβ signaling during iL3 arrest and subsequent downregulation during iL3 activation may have been an important step for the evolution of nematode parasitism. While there appears to be only a single dauer TGFβ ligand in the clade V/clade 9 nematodes *N. brasiliensis* (this study), *H. contortus* [49], *A. caninum* [24], *N. americanus* [69], as well as *C. elegans* [56], there have been several duplications of the genes encoding these ligands in clade IV/clade 10 parasitic nematodes [39]. In future studies examining genetic changes accompanying the evolution of parasitism, it may be informative to investigate when these changes in dauer TGFβ signaling arose.

Since dafachronic acid activates the *N. brasiliensis* DAF-12 homolog, we sought to determine the enzyme(s) responsible for endogenous dafachronic acid production in *N. brasiliensis* by examining the regulation of each cytochrome P450-encoding transcript during iL3 activation with either temperature or Δ7-dafachronic acid. Since *Cel-daf-9* is regulated by a positive feedback loop [61], we hypothesized that the cytochrome P450 responsible for dafachronic acid production in *N. brasiliensis* would be similarly upregulated by temperature and dafachronic acid. We identified several cytochrome P450-encoding transcripts, including a transcript encoding *Nbr*-CYP22A1 that phylogenetically groups with *Cel*-DAF-9, that are indeed upregulated during temperature- and dafachronic acid-mediated iL3 activation in *N. brasiliensis*. Although we cloned several of these transcripts, we were unable to determine which cytochrome P450 is responsible for dafachronic acid production in the parasite due to our inability to express these proteins *in vitro*. Similar studies in *S. stercoralis* have also been hampered by difficulties with parasite cytochrome P450 expression in HEK293 cells despite codon optimization; future studies to identify the DAF-9-like enzyme in parasitic nematodes may need to seek alternative strategies.

Since *N. brasiliensis* iL3 can be activated solely by host-like temperature, we sought to determine whether dafachronic acid can fully recapitulate temperature-dependent activation. Regardless of culture temperature, iL3 activated in 20 °C, 26 °C, and 37 °C dafachronic acid conditions broadly have similar transcript abundances for many of the dauer pathway genes as iL3 activated in the 37 °C vehicle control. These observations suggest that DAF-12-mediated signaling may include feed-back/feed-forward loops to ensure the full iL3 activation program is instituted in the presence of the ligand.

While genetic studies place DAF-12 downstream of IIS in *C. elegans* [15], the relationship between these two pathways in hookworms and other parasitic nematodes is less clear. In *A. caninum*, addition of Δ7-dafachronic acid fails to rescue inhibition of feeding by the IIS inhibitor LY294002 [23], and similar results have been observed in *S. stercoralis* [44]. These data, suggesting that DAF-12 signaling may regulate IIS, are bolstered by our observation that addition of dafachronic acid modulates ILP-encoding transcripts in *N. brasiliensis*. Similar regulation of ILP-encoding transcripts by dafachronic acid has also been observed in *S. stercoralis* [44]. Together, these observations point to a more complex relationship between the DAF-12 signaling and IIS pathways during the activation of iL3 in parasitic nematodes. Future studies examining the gene expression profile of iL3 activated inside a permissive host to iL3 activated *in vitro* with temperature and/or dafachronic acid at multiple time points may illuminate differences in gene expression between these modes of iL3 activation.

## Conclusions

Together with studies in other parasitic nematodes, our data support that at least two of four canonical dauer pathways indeed regulate iL3 activation in hookworms. Since iL3 can undergo arrest inside the human host [70], an understanding of the genetic pathways controlling their activation may provide insight into development of future therapeutic strategies. As dafachronic acid can reduce worm burden in other species of parasitic nematodes [71], similar examination of dafachronic acid as a therapeutic agent in hookworm infections may be a fruitful area of future study.

## Supplementary information

**Additional file 1: Data S1.***Nippostrongylus brasiliensis* dauer homolog transcript coding sequences.

**Additional file 2: Data S2.***Nippostrongylus brasiliensis* dauer homolog genome annotations.

**Additional file 3: Data S3.***Nippostrongylus brasiliensis* dauer homolog predicted protein sequences.

**Additional file 4: Data S4.** Primer sequences.

**Additional file 5: Data S5.***Nippostrongylus brasiliensis* StringTie-predicted transcript genome annotations.

**Additional file 6: Data S6.***Nippostrongylus brasiliensis* StringTie-predicted EdgeR-normalized transcript counts.

**Additional file 7: Data S7.***Nippostrongylus brasiliensis* EdgeR differentially expressed transcripts.

**Additional file 8: Table S1.** Dafachronic acid stimulates resumption of feeding in *N. brasiliensis* iL3 at non-permissive temperatures.

**Additional file 9: Figure S1.** Regulation of transcripts encoding cGMP signaling pathway components during *N. brasiliensis* iL3 activation.

**Additional file 10: Figure S2.** Regulation of transcripts encoding IIS pathway components during *N. brasiliensis* iL3 activation.

**Additional file 11: Figure S3.***Nbr*-CYP-22A1 groups with *Cel*-DAF-9 by phylogenetic analysis.

**Additional file 12: Data S8.***Nippostrongylus brasiliensis* cytochrome P450-encoding transcript abundance profiles.

**Additional file 13: Figure S4.** Regulation of transcripts encoding components of a proposed dafachronic acid biosynthetic pathway during *N. brasiliensis* iL3 activation.

## Data Availability

The datasets supporting the conclusions of this article are available in the NCBI repository under BioProject ID PRJNA574186.

## Abbreviations

cGMP: cyclic guanosine monophosphate
CPM: counts per million reads mapped
GPCR: G protein-coupled receptor
HA: hemagglutinin
HEK: human embryonic kidney
IGF-1: insulin-like growth factor 1
IIS: insulin/insulin growth factor 1 signaling
iL3: infectious third-stage larvae
ILP: insulin-like peptide
PCR: polymerase chain reaction
RLU: relative luciferase units
RPMI: Roswell Park Memorial Institute
SEM: standard error of the mean
TGFβ: transforming growth factor beta
TMM: trimmed mean of M
