# Supplementary material for: Dafachronic acid and temperature regulate canonical dauer pathways during Nippostrongylus brasiliensis infectious larvae activation
Source: Parasit Vectors. 2020 Apr 1;13:162. doi: 10.1186/s13071-020-04035-z (PMC7110753; doi:10.1186/s13071-020-04035-z)
Supplement: Supplementary file 8 — Additional file 8: Table S1. Dafachronic acid stimulates resumption of feeding in N. brasiliensis iL3 at non-permissive temperatures. [file 13071_2020_4035_MOESM8_ESM.docx]

**Additional File 8: Table S1. Dafachronic acid stimulates resumption of feeding in *N. brasiliensis* iL3 at non-permissive temperatures.**

| Incubation temperature and treatment | Percent iL3 feeding after 24 hours | | | | |
| --- | --- | --- | --- | --- | --- |
|  | **Replicate 1** | **Replicate 2** | **Replicate 3** | **Replicate 4** | **Mean** |
| 22 °C |  |  |  |  |  |
| Vehicle (ethanol) | 1.2 % | 0.0 % |  |  | 0.6 % |
| Δ7-dafachronic acid [10 μM] | 92.0 % | 93.8 % |  |  | 92.9 % |
|  |  |  |  |  |  |
| 26 °C |  |  |  |  |  |
| Vehicle (ethanol) | 0.0 % | 0.0 % | 0.6 % | 0.0 % | 0.1 % |
| Δ7-dafachronic acid [0.5 μM] | 19.3 % |  |  | 15.9 % | 17.6 % |
| Δ7-dafachronic acid [1 μM] | 65.0 % |  | 86.0 % | 61.5 % | 70.8 % |
| Δ7-dafachronic acid [10 μM] | 92.2 % | 96.3 % | 95.6 % | 94.2 % | 94.6 % |
|  |  |  |  |  |  |
| 37 °C |  |  |  |  |  |
| Vehicle (ethanol) | 97.4 % |  |  |  |  |
| Δ7-dafachronic acid [10 μM] | 98.4 % |  |  |  |  |
