# Supplementary material for: Dafachronic acid and temperature regulate canonical dauer pathways during Nippostrongylus brasiliensis infectious larvae activation
Source: Parasit Vectors. 2020 Apr 1;13:162. doi: 10.1186/s13071-020-04035-z (PMC7110753; doi:10.1186/s13071-020-04035-z)
Supplement: Supplementary file 9 — Additional file 9: Figure S1. Regulation of transcripts encoding cGMP signaling pathway components during N. brasiliensis iL3 activation. [file 13071_2020_4035_MOESM9_ESM.pdf]

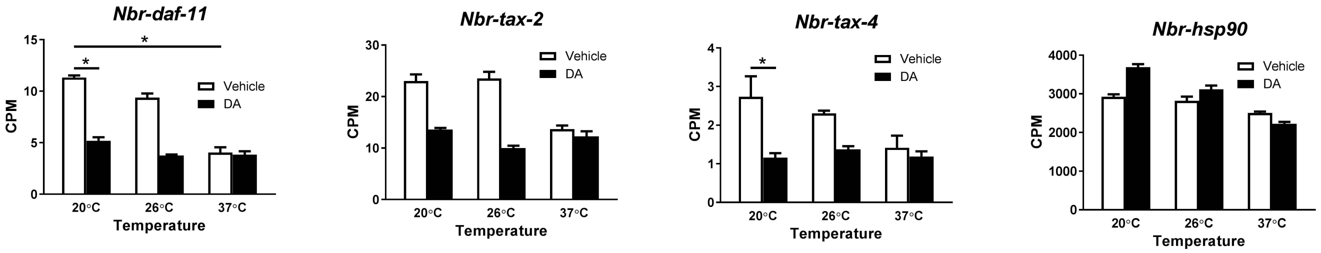

Figure S1. Regulation of transcripts encoding cGMP signaling pathway components during *N. brasiliensis* iL3 activation.

Transcript abundances were determined for the coding regions of the following genes predicted to constitute a signaling pathway regulated by cyclic guanosine monophosphate (cGMP): *Nbr-daf-11*, which is predicted to encode a guanylyl cyclase; *Nbr-tax-2* and *Nbr-tax-4*, which are predicted to encode the subunits of a cyclic nucleotide-gated cation channel; and *Nbr-hsp90*, which is predicted to encode a homolog of *Cel-hsp90* (formerly *Cel-daf-21*). *N. brasiliensis* iL3 were treated with either Δ7-dafachronic acid (DA) or the vehicle control (ethanol) for 24 hours at 20 °C, 26 °C, or 37 °C. TMM-normalized transcript abundance was plotted as the mean counts per million (CPM) for each condition; error bars represent the SEM. Statistical significance was evaluated between 20 °C vehicle and 20 °C DA conditions and also between 20 °C vehicle and 37 °C vehicle conditions; * = fold-change >2.0, FDR-adjusted p-value <0.05.
