# Supplementary material for: Dafachronic acid and temperature regulate canonical dauer pathways during Nippostrongylus brasiliensis infectious larvae activation
Source: Parasit Vectors. 2020 Apr 1;13:162. doi: 10.1186/s13071-020-04035-z (PMC7110753; doi:10.1186/s13071-020-04035-z)
Supplement: Supplementary file 11 — Additional file 11: Figure S3.Nbr-CYP-22A1 groups with Cel-DAF-9 by phylogenetic analysis. [file 13071_2020_4035_MOESM11_ESM.pdf]

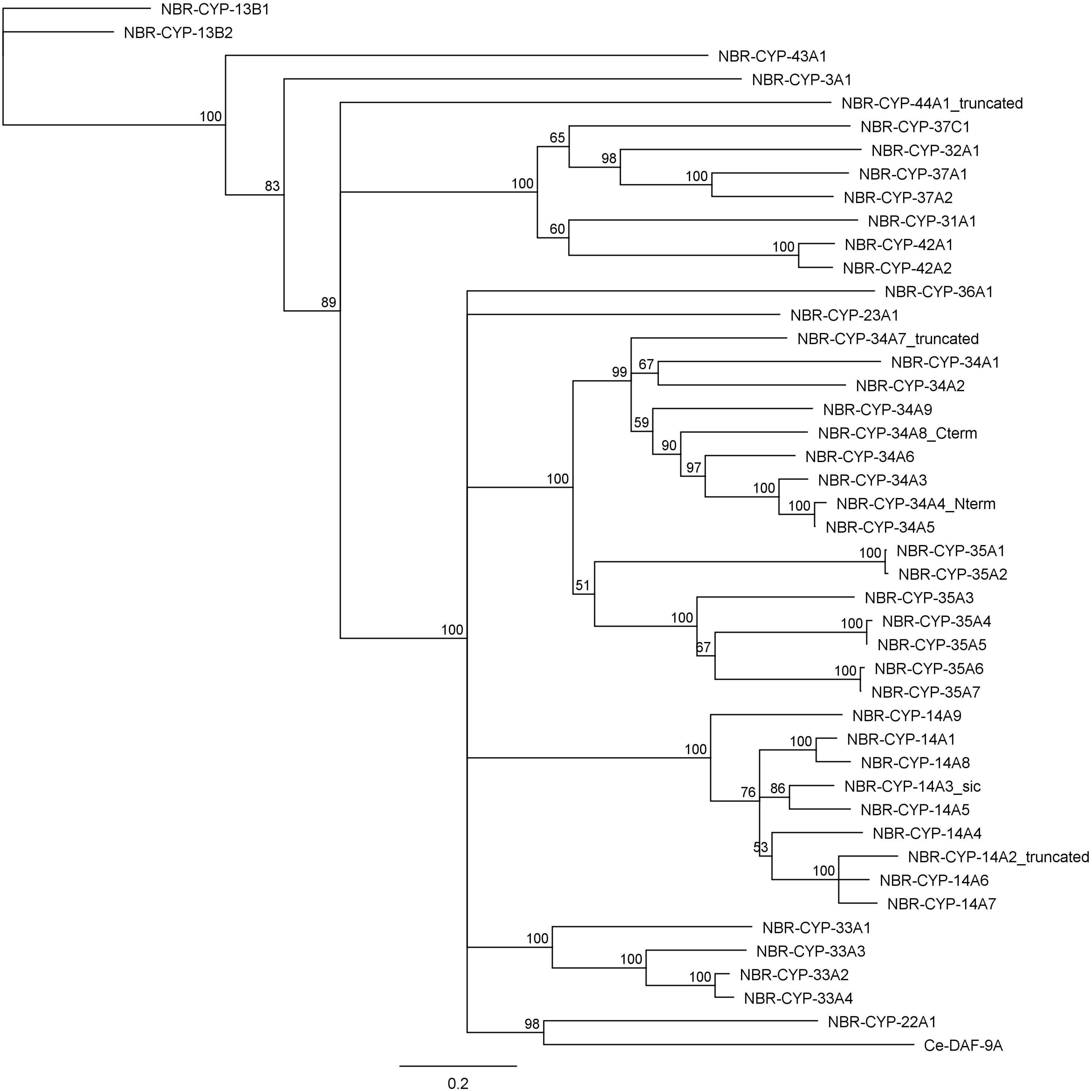

Figure S3. *Nbr*-CYP-22A1 groups with *Cel*-DAF-9 by phylogenetic analysis. *N. brasiliensis* transcripts predicted to encode polypeptides with a cytochrome P450 domain were translated; predicted protein sequences were aligned, along with *C. elegans* DAF-9A, using ClustalW. A neighbor-joining phylogenetic tree was constructed with 100 iterations of bootstrapping. *Nbr*-CYP-22A1 grouped most closely with *Cel*-DAF-9. Scale bar represents number of substitutions per position.
