# Supplementary material for: Dafachronic acid and temperature regulate canonical dauer pathways during Nippostrongylus brasiliensis infectious larvae activation
Source: Parasit Vectors. 2020 Apr 1;13:162. doi: 10.1186/s13071-020-04035-z (PMC7110753; doi:10.1186/s13071-020-04035-z)
Supplement: Supplementary file 12 — Additional file 12: Data S8.Nippostrongylus brasiliensis cytochrome P450-encoding transcript abundance profiles. [file 13071_2020_4035_MOESM12_ESM.pdf]

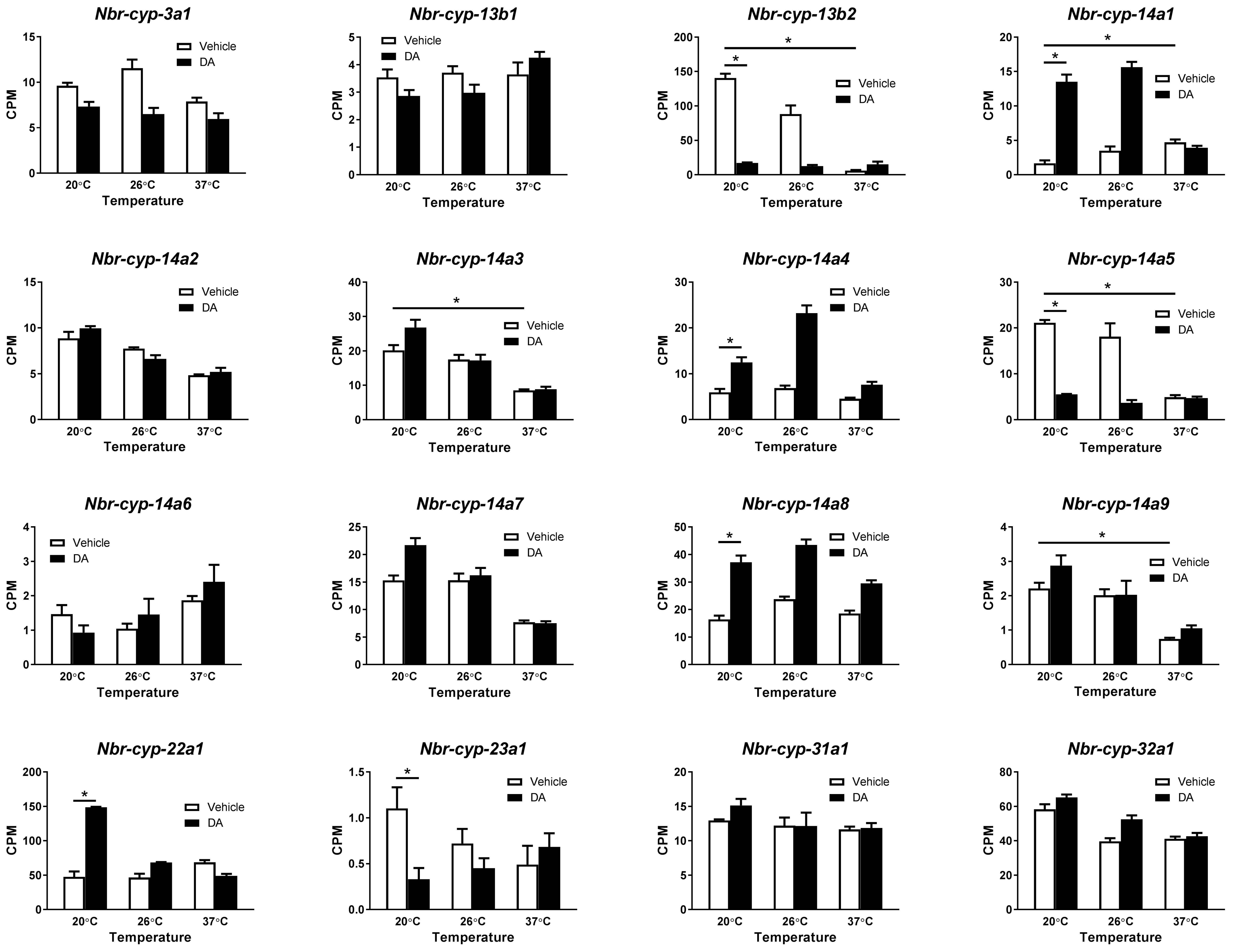

Nbr-cyp-3a1
15
10
5
0
CPM
Vehicle
DA
20°C
26°C
37°C
Temperature
Nbr-cyp-13b1
5
4
3
2
1
0
CPM
Vehicle
DA
20°C
26°C
37°C
Temperature
Nbr-cyp-13b2
200
150
100
50
0
CPM
*
*
Vehicle
DA
20°C
26°C
37°C
Temperature
Nbr-cyp-14a1
20
15
10
5
0
CPM
*
*
Vehicle
DA
20°C
26°C
37°C
Temperature
Nbr-cyp-14a2
15
10
5
0
CPM
Vehicle
DA
20°C
26°C
37°C
Temperature
Nbr-cyp-14a3
40
30
20
10
0
CPM
*
Vehicle
DA
20°C
26°C
37°C
Temperature
Nbr-cyp-14a4
30
20
10
0
CPM
*
Vehicle
DA
20°C
26°C
37°C
Temperature
Nbr-cyp-14a5
30
20
10
0
CPM
*
*
Vehicle
DA
20°C
26°C
37°C
Temperature
Nbr-cyp-14a6
4
3
2
1
0
CPM
Vehicle
DA
20°C
26°C
37°C
Temperature
Nbr-cyp-14a7
25
20
15
10
5
0
CPM
Vehicle
DA
20°C
26°C
37°C
Temperature
Nbr-cyp-14a8
50
40
30
20
10
0
CPM
*
Vehicle
DA
20°C
26°C
37°C
Temperature
Nbr-cyp-14a9
4
3
2
1
0
CPM
*
Vehicle
DA
20°C
26°C
37°C
Temperature
Nbr-cyp-22a1
200
150
100
50
0
CPM
*
Vehicle
DA
20°C
26°C
37°C
Temperature
Nbr-cyp-23a1
1.5
1.0
0.5
0.0
CPM
*
Vehicle
DA
20°C
26°C
37°C
Temperature
Nbr-cyp-31a1
20
15
10
5
0
CPM
Vehicle
DA
20°C
26°C
37°C
Temperature
Nbr-cyp-32a1
80
60
40
20
0
CPM
Vehicle
DA
20°C
26°C
37°C
Temperature

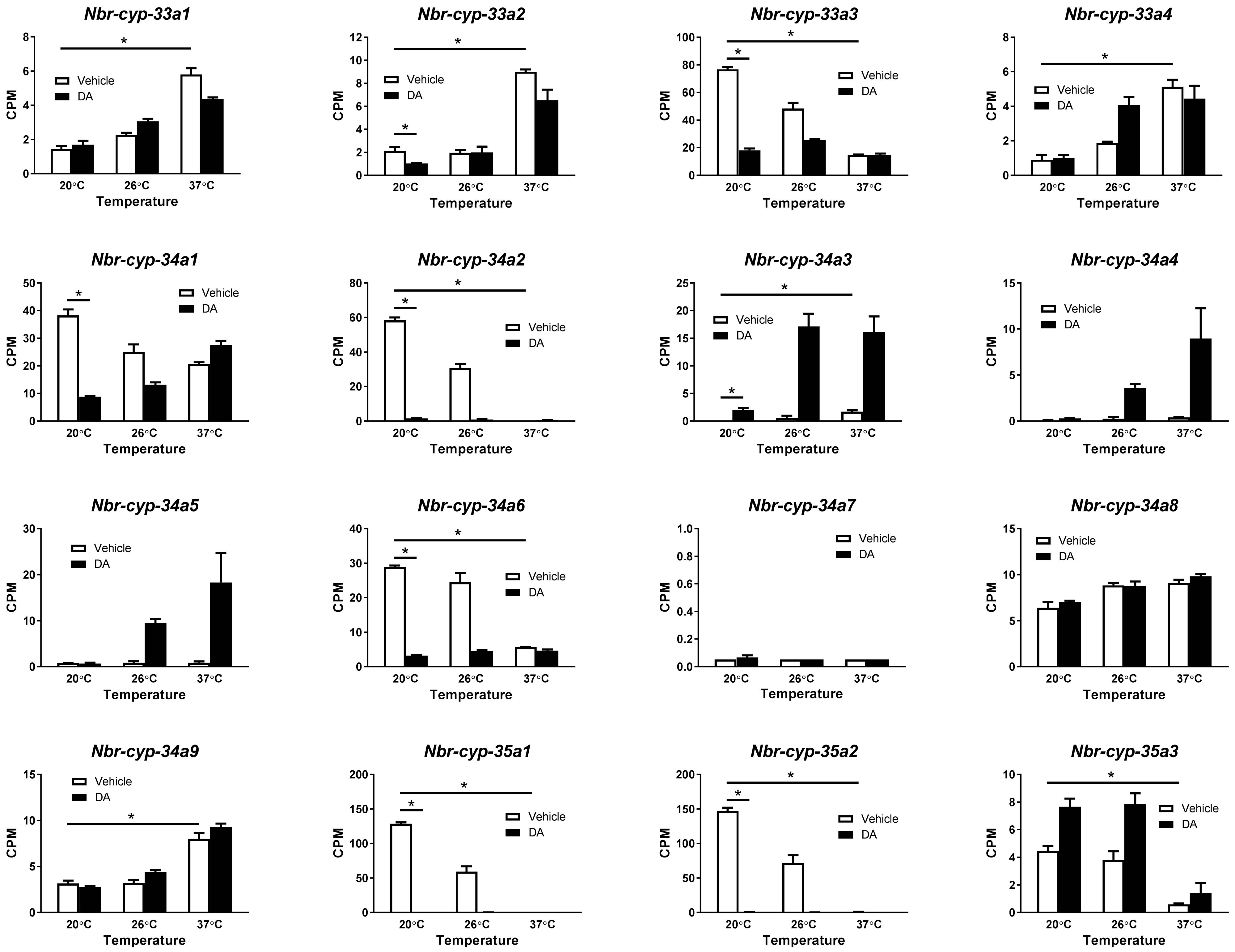

Nbr-cyp-33a1
Nbr-cyp-33a2
Nbr-cyp-33a3
Nbr-cyp-33a4
Nbr-cyp-34a1
Nbr-cyp-34a2
Nbr-cyp-34a3
Nbr-cyp-34a4
Nbr-cyp-34a5
Nbr-cyp-34a6
Nbr-cyp-34a7
Nbr-cyp-34a8
Nbr-cyp-34a9
Nbr-cyp-35a1
Nbr-cyp-35a2
Nbr-cyp-35a3
CPM
Vehicle
DA
20°C
26°C
37°C
Temperature
*

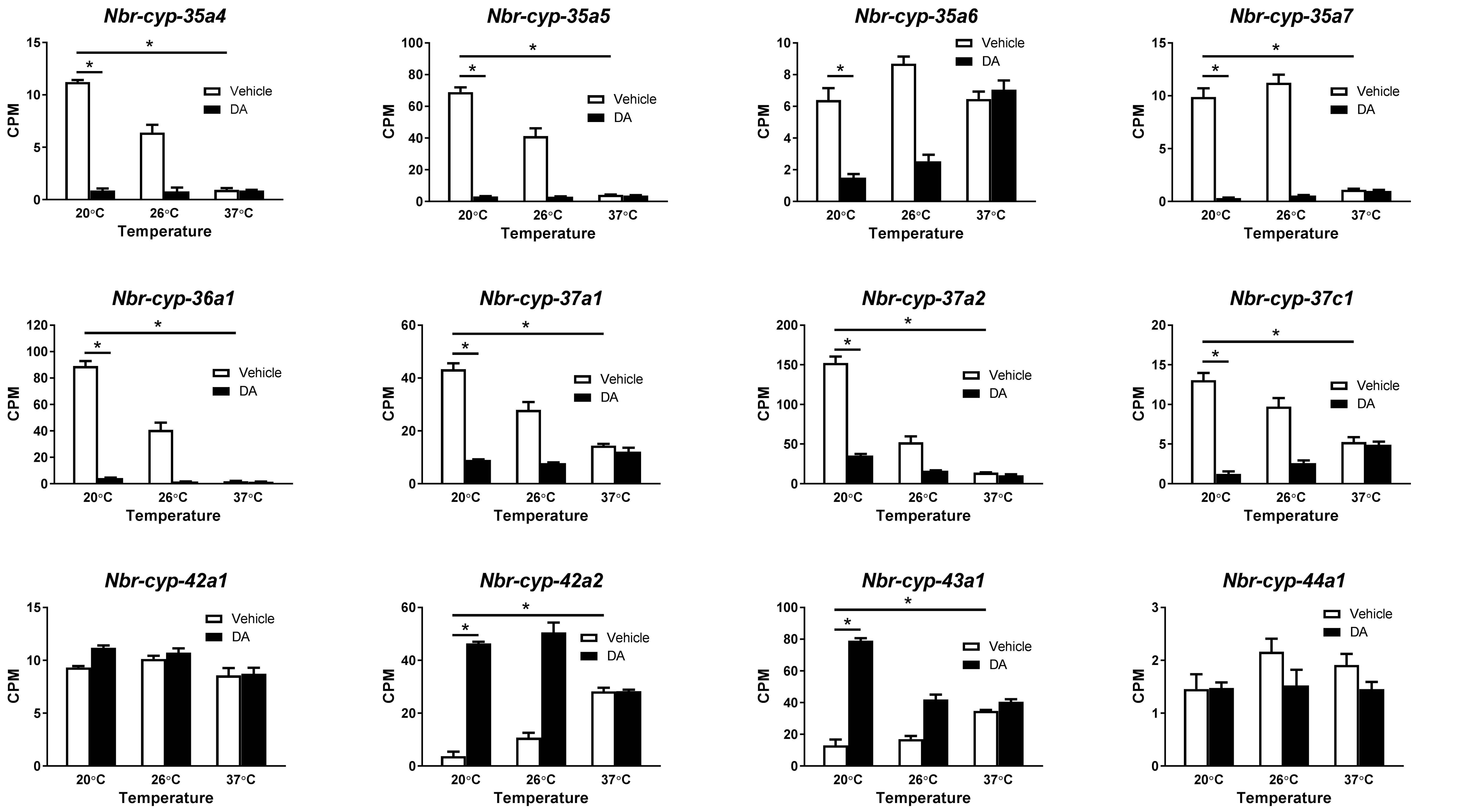

Data S8 *Nippostrongylus brasiliensis* cytochrome P450-encoding transcript abundance profiles.
Transcript abundances were determined for the coding regions of genes predicted to encode cytochrome P450s in *N. brasiliensis*. *N. brasiliensis* iL3 were treated with either Δ7-dafachronic acid (DA) or the vehicle control (ethanol) for 24 hours at 20 °C, 26 °C, or 37 °C. TMM-normalized transcript abundance was plotted as the mean counts per million (CPM) for each condition; error bars represent the SEM. Statistical significance was evaluated between 20 °C vehicle and 20 °C DA conditions and also between 20 °C vehicle and 37 °C vehicle conditions; * = fold-change >2.0, FDR-adjusted p-value <0.05.
