# Supplementary material for: Dafachronic acid and temperature regulate canonical dauer pathways during Nippostrongylus brasiliensis infectious larvae activation
Source: Parasit Vectors. 2020 Apr 1;13:162. doi: 10.1186/s13071-020-04035-z (PMC7110753; doi:10.1186/s13071-020-04035-z)
Supplement: Supplementary file 13 — Additional file 13: Figure S4. Regulation of transcripts encoding components of a proposed dafachronic acid biosynthetic pathway during N. brasiliensis iL3 activation. [file 13071_2020_4035_MOESM13_ESM.pdf]

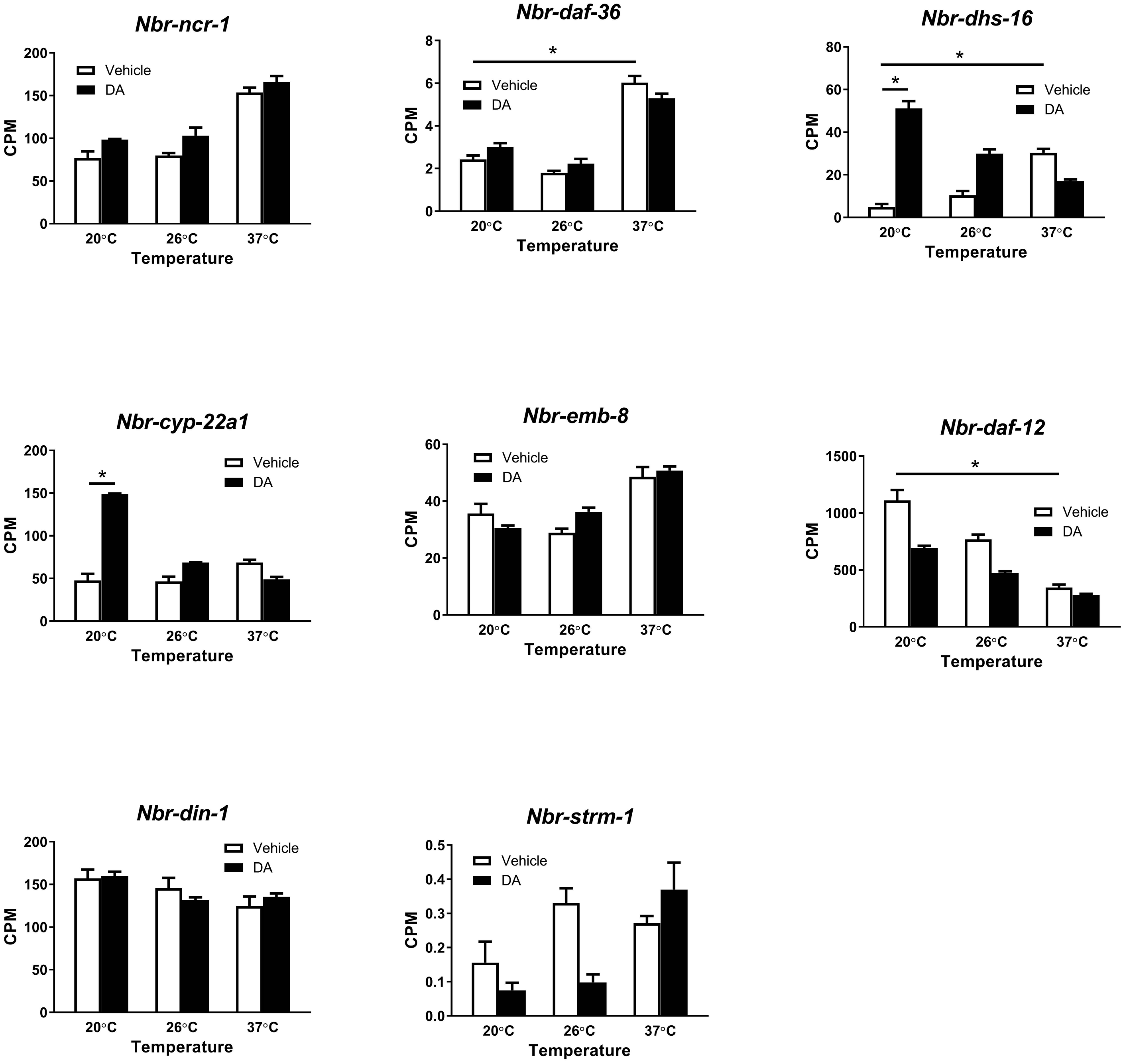

Figure S4. Regulation of transcripts encoding components of a proposed dafachronic acid biosynthetic pathway during *N. brasiliensis* iL3 activation.

Transcript abundances were determined for the coding regions of genes predicted to compose a dafachronic acid biosynthesis pathway in *N. brasiliensis*. *N. brasiliensis* iL3 were treated with either Δ7-dafachronic acid (DA) or the vehicle control (ethanol) for 24 hours at 20 °C, 26 °C, or 37 °C. TMM-normalized transcript abundance was plotted as the mean counts per million (CPM) for each condition; error bars represent the SEM. Statistical significance was evaluated between 20 °C vehicle and 20 °C DA conditions and also between 20 °C vehicle and 37 °C vehicle conditions; * = fold-change >2.0, FDR-adjusted p-value <0.05.
